# Germline *BRCA1/2* Variants in Polish Patients with Family History of Breast and Ovarian Cancer: Prevalence, CNV Detection, and Identification of a Novel Loss-of-Function Mutation

**DOI:** 10.3390/curroncol33010010

**Published:** 2025-12-24

**Authors:** Sebastian Skoczylas, Tomasz Płoszaj, Izabela Dróżdż, Hanna Moczulska, Marcin Serafin, Katarzyna Piekarska, Olga Wojtyczka, Karolina Żeżawska, Agnieszka Zmysłowska

**Affiliations:** 1 Department of Clinical Genetics, Medical University of Lodz, 92-213 Lodz, Poland; tomasz.ploszaj@umed.lodz.pl (T.P.);; 2 Student Society for Genetic Counselling and Clinical Genetics at the Department of Clinical Genetics, Medical University of Lodz, 92-213 Lodz, Poland

**Keywords:** NGS, *BRCA1*, *BRCA2*, CNV, breast cancer, ovarian cancer, familial risk

## Abstract

Background/Objectives: Pathogenic and likely pathogenic variants in the *BRCA1* and *BRCA2* genes are associated with a significantly increased risk of breast and/or ovarian cancer. We investigated genetic variants in a cohort of 450 unaffected individuals with a family history of breast and/or ovarian cancer, involving at least one first-degree relative. Methods: Next-generation sequencing (NGS) was used to analyze the coding regions of these two genes, with copy number variation (CNV) analysis. Results: A total of 16 unique to our cohort variants classified as pathogenic or likely pathogenic were identified in 22 patients, including one novel loss-of-function variant in *BRCA1* gene. Furthermore, we identified a deletion of exon 21 in the *BRCA1* gene in two patients. Conclusions: These results emphasize the difficulties involved in molecular diagnostics and indicate the need for further research into new predictive models for patients with hereditary breast and ovarian cancer.

## Simple Summary

Among the Polish population, around 4% of breast cancer patients and 10% of ovarian cancer patients have pathogenic variants of the *BRCA1* gene, whereas variants of the *BRCA2* gene are uncommon. Short-read sequencing provides a comprehensive range of data, including the detection of CNVs (copy number variants), but it still poses challenges when using standard NGS workflows. In this study, we investigated genetic variants in a cohort of 450 unaffected individuals with a family history of breast and/or ovarian cancer, involving at least one first-degree relative, with particular emphasis on CNV detection. In our study, the detection accuracy was just 4.89%, indicating that the genetic predisposition to cancer could not be demonstrated in the majority of patients.

## 1. Introduction

The role of predisposition to breast and ovarian cancer caused by germline pathogenic variants in *BRCA1* and *BRCA2* is well documented in the literature. Pathogenic variants in the *BRCA1* gene increase the risk of developing breast cancer to 55–72%, whereas variants in *BRCA2* increase the risk to 45–69% [1]. In the Polish population, about 4% of breast cancer patients and 10% of ovarian cancer patients carry pathogenic variants in the *BRCA1* gene [2,3,4]. According to the Szwiec et. al. paper, Polish women diagnosed with breast cancer below the age of 50 should be screened with a panel of six founder mutations in *BRCA1*: NM_007294.4:c.181T>G, NM_007294.4:c.4035del, NM_007294.4:c.5266dup, NM_007294.4:c.3700_3704del, NM_007294.4:c.68_69del and NM_007294.4:c.5251C>T (C61G, 4153delA, 5382insC, 3819del5, 185delAG and 5370C>T) 4) [4]. The screening used to include these variants, but now the standard testing includes targeted NGS of *BRCA1/2 genes*.

Another important aspect is the care of relatives of individuals with a history of breast and/or ovarian cancer, which may improve predictive accuracy in the case of hereditary variants present in the family [1]. While *BRCA1* and *BRCA2* are the primary genes associated with hereditary breast and ovarian cancer, several other high-penetrance genes (e.g., *TP53*, *PTEN*, *CDH1*) and moderate-risk genes (e.g., *ATM*, *CHEK2*, *BARD1*, etc.) also contribute to cancer susceptibility [1,5,6,7].

Why is testing for variants in the *BRCA1/2* genes so crucial? Individuals who carry germline pathogenic variants in these genes are sensitive to poly(ADP-ribose) polymerase (PARP) inhibitors and DNA-damaging agents, including platinum-based chemotherapies. Therefore, recognizing them is important, as this influences both therapeutic and preventive strategies. This information is used to determine eligibility for targeted therapies, which have been shown to provide this group with significant clinical benefits [8]. In addition to their therapeutic value, *BRCA1/2* tests play a key role in preventing cancer and managing family risk. This allows for the implementation of personalized surveillance programs, including earlier and more frequent screening tests, such as magnetic resonance imaging, ultrasound and mammography. Importantly, testing is beneficial not only in families with a high risk of cancer, but also in those with a moderate or unclear family history of breast and ovarian cancer [1]. Some carriers may not meet the classic criteria [9]. They can still benefit from preventive interventions once identified. Furthermore, relatives can be tested to help identify those who are unaware of their hereditary predisposition to cancer [10]. This enables early intervention before the disease develops, before any symptoms appear [11]. In this context, genetic testing is becoming the basis for personalized medicine and cancer prevention at a population level. It also facilitates cascade testing of relatives, promoting early detection in asymptomatic carriers and enabling timely medical interventions, even in families without a strong cancer history [9].

Given the large number of variants in the *BRCA1/2* genes, molecular diagnostics in breast cancer patients should be performed using next-generation sequencing (NGS) [1,5,6,7]. Despite the fact that short read sequencing offers a comprehensive range of data from sequencing, even for deep intronic variants or CNVs (copy number variants), it still poses a challenge when using standard NGS workflows [12]. Despite the widespread use of NGS in clinical practice, the prevalence and spectrum of *BRCA1/2* variants, particularly CNVs, in Polish families with unaffected individuals and a family history of breast and/or ovarian cancer involving at least one first-degree relative, remain limited.

This study aimed to expand current knowledge of germline *BRCA1/2* variants in the Polish population by analyzing patients with a family history of breast and/or ovarian cancer, with particular focus on the detection of copy number variants (CNVs).

## 2. Materials and Methods

A total of 450 unaffected individuals (9.11% male and 90.89% female) with a family history of breast and/or ovarian cancer were recruited between 2021 and 2024 at the Clinical Genetics Outpatient Clinic of the Department of Clinical Genetics at the Medical University of Lodz, Poland. In 2021, within the Polish Ministry of Health program, 12,277 individuals underwent *BRCA1* and *BRCA2* genetic testing, including 1873 analysis using NGS [13]. All participants underwent pedigree analysis and medical examination. The criterion for inclusion in the study group was the presence of at least one first-degree relative with breast or ovarian cancer. Participants who had already been diagnosed with either of these cancers or who had undergone further genetic testing since the initial *BRCA1/2* analysis and obtained a positive result were excluded. Thus, the analyzed cohort consisted exclusively of unaffected individuals at increased familial risk. Peripheral blood was collected from participants in the study group after they had given their informed written consent to genetic testing.

DNA was extracted from peripheral blood using the Maxwell^®^ RSC Instrument (Maxwell^®^ RSC Blood DNA Kit, Promega, Madison, WI, USA). Libraries were prepared using the Devyser BRCA NGS kit (Devyser, Stockholm, Sweden) according to the manufacturer’s instructions. Paired-end sequencing (2 × 149 bp) was performed on a MiSeq System (Illumina). The sequencing quality was assessed using the manufacturer’s software. The mean sequencing coverage was 930x for both *BRCA1* and *BRCA2*. Samples that did not pass quality control were sequenced to obtain the required parameters. Supplementary Figure S1 provides a detailed visualization of exon-by-exon coverage (minimal, mean and maximal) for both *BRCA1* and *BRCA2*.

CNV confirmation was performed using MLPA (Multiplex Ligation-dependent Probe Amplification (SALSA^®^ MLPA^®^ Probemix P002 *BRCA1*, MRC Holland, Amsterdam, The Netherlands) according to the manufacturer’s instructions. Only CNVs confirmed by MLPA were reported.

The FASTQ file was analyzed using Amplicon Suite 3.7.0 (SmartSeq), and for additional variant annotation and variant selection, we used local ANNOVAR (2019Oct24) [9]. The variants to be analyzed were prioritized according to the ClinVar prediction, the loss-of-function character, and finally the frequency in the GnomAD v4.1 reference population [14], which is below 0.01. For the analysis of CNVs from the *BRCA1* and *BRCA2* genes, dedicated software was applied. All statistical analyses were performed using Python (version 3.11) and the libraries NumPy [10] and SciPy [11]. The odds ratio (OR), 95% confidence intervals and *p*-values were calculated using a two-sided Fisher’s exact test based on 2 × 2 contingency tables.

## 3. Results

A total of 450 (*n* = 409 female, *n* = 41 male) unaffected individuals were included in the study group. CNV analysis was possible in only 270 patients due to data quality constraints. Detailed characteristics are shown in Table 1.

**Figure 1:**
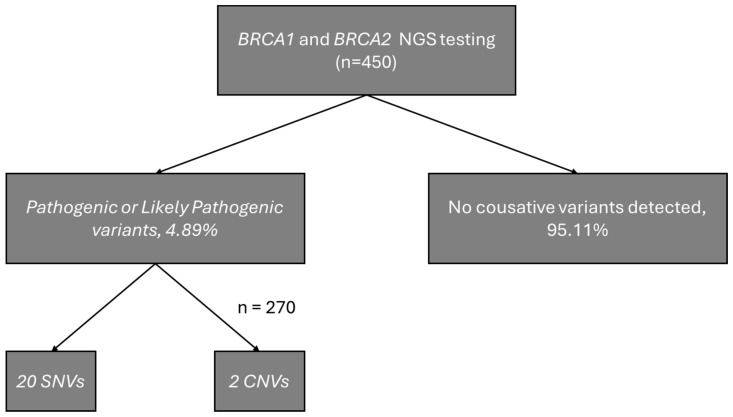
Schematic diagram of the obtained results.

**Figure 2:**
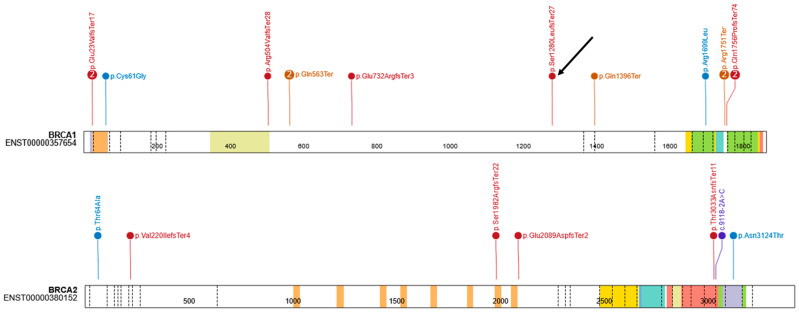
Distribution of single-nucleotide variants in the study. The arrow indicates the novel variant.

A total of 22 individuals (4.89%) were identified as carriers of pathogenic or likely pathogenic variants, including 16 carriers of *BRCA1* variants (10 unique variants) and 6 carriers of *BRCA2* variants (6 unique variants). Among the identified variants, 14 were classified as pathogenic by the ClinGen Expert Panel and 3 as pathogenic or likely pathogenic according to ClinVar (one of our records). The carrier frequency of *BRCA1* was significantly higher in the study group than in gnomAD v4.1 (OR = 23.17; 95% CI: 13.99–38.40; *p* = 1.07 × 10^−16^), whereas no significant difference was observed for *BRCA2* (OR = 1.88; 95% CI: 0.84–4.20; *p* = 0.15). Odds ratios were calculated relative to the gnomAD v4.1 European (non-Finnish) population. Figure 1 presents a schematic diagram of the obtained results. Founder mutations were observed in 7 patients, and 4 unique variants were identified. The distribution of single-nucleotide variants in the study cohort is shown in Figure 2, visualized using ProteinPaint (https://proteinpaint.stjude.org, accessed on 17 December 2025) [15].

Table 2 presents the selected variants with HGVS nomenclature, their classification and study group frequency, and the predicted protein changes.

One novel variant was identified in the *BRCA1* gene (NM_007294.4:c.3837del; NP_009225.1:p.(Ser1280LeufsTer27), classified as likely pathogenic and submitted to ClinVar (VCV003384156.1). Additionally, twelve missense variants were identified, all of which had a minor allele frequency of less than 0.01 in gnomAD v4.1. Ten of these variants have conflicting interpretations in ClinVar. One variant had a VUS classification (NM_007294.4: c.1844C>A; p.(Ser615Tyr); VCV002811842.2), and the remaining variant without classification was NM_000059.4:c.190A>G p.(Thr64Ala). They were both evaluated according to the ACMG [16] and ClinGen *BRCA1/2* VCEP classifications [17], and were classified as likely benign. According to the AlphaMissense [18], NM_007294.4:c.1844C>A was classified as likely benign (score: 0.119), while BRCA2:c.190A>G was classified as ambiguous (score: 0.38). Additionally, the lack of segregation was another aspect of the final decision.

## 4. Discussion

Our study yielded a positive result in 4.89% of cases, of which 20 were SNV variants and two were CNV mutations. This result is slightly lower than the results of other studies conducted in Poland, in which individuals underwent *BRCA1/2* testing [2,3,4,19]. However, unlike previous studies that focused on affected patients, our cohort consisted entirely of unaffected individuals with a positive family history of breast and/or ovarian cancer.

Unlike *BRCA1* (*p* = 1.07 × 10^−16^), no significant difference in carrier frequency was observed for *BRCA2* (*p* = 0.15) when compared with gnomAD v4.1. This finding is consistent with a study by Cybulski et al., which found that variants in *BRCA2* are relatively rare in familial breast cancer in Poland [20]. Consequently, the absence of statistical significance of variants for the *BRCA2* gene in our dataset should be interpreted with caution, particularly in the context of population structure, penetrance differences and cohort selection. Furthermore, the insignificant *p*-value for *BRCA2* can partly be explained by the small number of observed carriers, which reduces the accuracy of the odds ratio estimate. This limitation is inherent in analyses of rare variants, emphasizing the importance of larger sample sizes for reliably detecting moderate enrichment signals.

Furthermore, the NM_007294.4:c.5266dup variant, which is most strongly associated with breast and/or ovarian cancer in Poland, was not the most frequently observed variant in our cohort [2,3,4,19]. Nevertheless, several studies have discussed pathogenic variants in the *BRCA1* and *BRCA2* genes associated with reduced penetrance [21,22,23]. Finally, the inclusion of unaffected individuals in our cohort may explain the observed frequency deviation, as many of the most severely affected families were likely diagnosed earlier in other clinical centers. Thus, despite the relatively small size of the study group, our study provides additional data on a new loss-of-function variant in the *BRCA1* gene (NM_007294.4:c.3837del; p.(Ser1280Leufs*27); ACMG: PVS1, PM2) and other described variants. The NM_007294.4:c.3837del variant is located in exon 10 of the *BRCA1* gene. This variant was absent from the gnomAD v4.1 database. The serine cluster domain spans amino acids 1280–1524, and the variant is in position 1800 aa. Mutation of these serine residues has clinical implications and may affect the localization of *BRCA1* to sites of DNA damage and the function of the DNA damage response [24].

It is worth noting that the process of detecting CNVs can be a difficult undertaking [12,25,26]. Identifying even a single CNV can have a significant impact on the further care of the patient and their family. Therefore, it is extremely important to accurately determine the exact diagnosis. Several technical challenges were encountered during CNV assessment using the Devyser BRCA NGS kit (NGS amplicon-based) in our study. Although the DNA quality and sequencing metrics (including coverage depth and Q30) were within acceptable ranges, a subset of the samples generated could not be analyzed for CNVs. We attempted to analyze DNA isolate purity parameters based on the A260/A280 spectrophotometric parameters, but this did not show a correlation with the success rate of CNV analysis from NGS data. Neither DNA concentration alone nor different reagent batches from different manufacturers had a significant impact on the success rate of CNV analysis. Consequently, it was not possible to evaluate certain samples for CNVs with confidence, which highlights the need for MLPA validation for negative samples. NGS kits based on probe enrichment may yield more reliable and reproducible CNV analysis results than those based on amplicon technology.

Given the growing number of available genetic test results, it is crucial to collect data on rare genetic variants across multiple families. These efforts could potentially lead to more reliable classification of variants, as demonstrated in the article by Caputo et al. [27]. In this context, the systematic collection of data from cohorts of unaffected individuals with a positive family history, as in the present study, can contribute to the future development of reference datasets for interpreting *BRCA1/2* variants in the Polish population. However, several methodological limitations should be taken into consideration. First of all, this study did not analyze deep intronic sequences, meaning that pathogenic variants in these locations of the genome could not be detected. Furthermore, not all patients had CNVs analyzed. Secondly, the analysis only considered two genes and did not involve a broader genetic assessment (e.g., *PALB2*, *CHEK2*, *ATM, RAD51C* and *RAD51D*). This reflects the clinical and financial constraints of the testing strategy used during the study period, when *BRCA1/2* testing was the only approach to screening that was reimbursed in Poland.

## 5. Conclusions

In conclusion, our study expands the spectrum of known genetic variants with a newly identified loss-of-function variant of *BRCA1*, which highlights the importance of including CNV analysis in routine *BRCA1/2* screening. Identifying this variant shows that clinically relevant changes can be missed in families with no obvious history of the condition. Furthermore, failing to incorporate reliable CNV analysis into standard workflows in amplicon arrays can result in false-negative results. In such cases, diagnostics should be extended to include additional tests, such as MLPA.

## Figures and Tables

**Table 1:** Characteristics of the study group.

Parameter	*n* (%)	Positive (%)	Negative/VUS (%)
Total number of patients	450 (100.00)	22 (4.89)	427 (95.11)
Sex			
Female	409 (90.89)	20 (4.89)	389 (95.11)
Male	41 (9.11)	2 (4.88)	39 (95.12)
Age at enrollment			
Median	45	_	_
IQR	37–56	_	_
Family history:		_	_
At least one first-degree relative	450 (100)	_	_
Affected by breast cancer	446 (99.11)	21 (4.67)	425 (92.67)
Affected by ovarian cancer	4 (0.89)	1 (25)	3 (75)

**Table 2:** The frequency and classification of the identified *BRCA1* (NM_007294.4) and *BRCA2* (NM_000059.4) variants are shown sorted by cDNA position.

Genetic Variant	HGVSp	ClinGen *BRCA1/2* VCEP Classifications	ClinVar	Number of Patients Allele Count/Allele Number
NM_007294.4:c.68_69del *	p.(Glu23ValfsTer17)	Pathogenic	Pathogenicity reviewed by expert panel	2/900
NM_007294.4:c.181T>G *	p.(Cys61Gly)	Pathogenic	Pathogenicity reviewed by expert panel	1/900
NM_007294.4:c.1510delC	p.(Arg504ValfsTer28)	Pathogenic	Pathogenicity reviewed by expert panel	1/900
NM_007294.4:c.1687C>T	p.(Gln563Ter)	Pathogenic	Pathogenicity reviewed by expert panel	2/900
NM_007294.4:c.2193_2196delAGAA	p.(Glu732ArgfsTer3)	Pathogenic	Pathogenicity reviewed by expert panel	1/900
NM_007294.4:c.3837del	p.(Ser1280LeufsTer27)	Likely_pathogenic	Likely_pathogenic	1/900
NM_007294.4:c.4186C>T	p.(Gln1396Ter)	Pathogenic	Pathogenicity reviewed by expert panel	1/900
NM_007294.4:c.5096G>T	p.(Arg1699Leu)	Pathogenic	Likely pathogenic criteria provided, multiple submitters, no conflicts	1/900
NM_007294.4:c.5251C>T *	p.(Arg1751Ter)	Pathogenic	Pathogenicity reviewed by expert panel	2/900
NM_007294.4:c.5266dup *	p.(Gln1756ProfsTer74)	Pathogenic	Pathogenicity reviewed by expert panel	2/900
NM_007294.4:c.5333-36_5406+400del	p.?	Pathogenic	Pathogenicity reviewed by expert panel	2/900
NM_000059.4:c.658_659delGT	p.(Val220IlefsTer4)	Pathogenic	Pathogenicity reviewed by expert panel	1/900
NM_000059.4:c.5946delT	p.(Ser1982ArgfsTer22)	Pathogenic	Pathogenicity reviewed by expert panel	1/900
NM_000059.4:c.6267_6269delGCAinsC	p.(Glu2089AspfsTer2)	Pathogenic	Pathogenicity reviewed by expert panel	1/900
NM_000059.4:c.9118-2A>C	p.?	Pathogenic	Pathogenic criteria provided, single submitter	1/900
NM_000059.4:c.9097dupA	p.(Thr3033AsnfsTer11)	Pathogenic	Pathogenicity reviewed by expert panel	1/900
NM_000059.4:c.9371A>C	p.(Asn3124Thr)	Likely_pathogenic	Pathogenicity reviewed by expert panel	1/900

* Founder mutations.

## Data Availability

Data were generated from routine patient diagnostics at the Central Teaching Hospital of the Medical University of Lodz. The data presented in this study are available on request from the corresponding author.

## Supplementary Materials

The following supporting information can be downloaded at: https://www.mdpi.com/article/10.3390/curroncol33010010/s1. Figure S1: Distribution of coverage across exons of the BRCA1 and BRCA2 genes.
