# Supplementary material for: Germline BRCA1/2 Variants in Polish Patients with Family History of Breast and Ovarian Cancer: Prevalence, CNV Detection, and Identification of a Novel Loss-of-Function Mutation
Source: Curr Oncol. 2025 Dec 24;33(1):10. doi: 10.3390/curroncol33010010 (PMC12839688; doi:10.3390/curroncol33010010)
Supplement: Supplementary file 1 [file curroncol-33-00010-s001.zip › curroncol-3931343-supplementary.pdf]

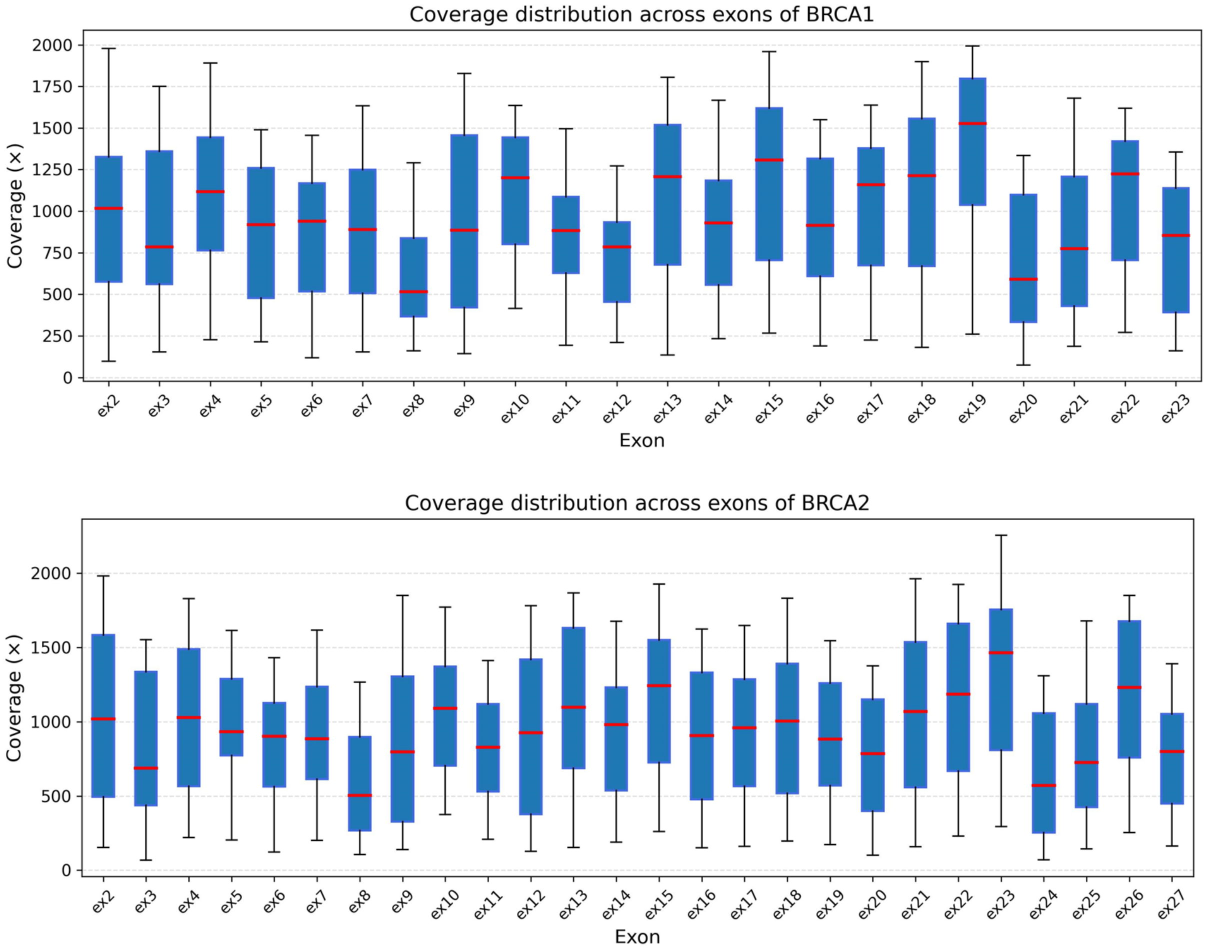

Supplementary Figure S1: Distribution of coverage across exons of the *BRCA1* and *BRCA2* genes. The horizontal red line shows the mean coverage, while the whiskers show the minimum and maximum coverage values.
